# The clinical and experimental treatment of Juvenile Idiopathic Arthritis

**DOI:** 10.1093/cei/uxad045

**Published:** 2023-04-19

**Authors:** L Nijhuis, J F Swart, B J Prakken, J van Loosdregt, S J Vastert

**Affiliations:** Center for Translational Immunology, University Medical Center Utrecht, Utrecht, The Netherlands; Department of pediatric rheumatology & immunology, University Medical Center Utrecht, Utrecht, The Netherlands; Department of pediatric rheumatology & immunology, University Medical Center Utrecht, Utrecht, The Netherlands; University of Utrecht, Utrecht, The Netherlands; Department of pediatric rheumatology & immunology, University Medical Center Utrecht, Utrecht, The Netherlands; University of Utrecht, Utrecht, The Netherlands; Center for Translational Immunology, University Medical Center Utrecht, Utrecht, The Netherlands; University of Utrecht, Utrecht, The Netherlands; Center for Translational Immunology, University Medical Center Utrecht, Utrecht, The Netherlands; Department of pediatric rheumatology & immunology, University Medical Center Utrecht, Utrecht, The Netherlands; University of Utrecht, Utrecht, The Netherlands

**Keywords:** juvenile idiopathic arthritis, translational immunology, treatment, mechanisms of disease

## Abstract

Juvenile idiopathic arthritis (JIA) is the most common chronic rheumatic disease in children and comprises of multiple subtypes. The most relevant disease subtypes, grouped upon current insight in disease mechanisms, are nonsystemic (oligo- and polyarticular) JIA and systemic JIA (sJIA). In this review, we summarize some of the main proposed mechanisms of disease in both nonsystemic and sJIA and discuss how current therapeutic modalities target some of the pathogenic immune pathways. Chronic inflammation in nonsystemic JIA is the result of a complex interplay between effector and regulatory immune cell subsets, with adaptive immune cells, specifically T-cell subsets and antigen-presenting cells, in a central role. There is, however, also innate immune cell contribution. SJIA is nowadays recognized as an acquired chronic inflammatory disorder with striking autoinflammatory features in the first phase of the disease. Some sJIA patients develop a refractory disease course, with indications for involvement of adaptive immune pathways as well. Currently, therapeutic strategies are directed at suppressing effector mechanisms in both non-systemic and sJIA. These strategies are often not yet optimally tuned nor timed to the known active mechanisms of disease in individual patients in both non-systemic and sJIA. We discuss current treatment strategies in JIA, specifically the ‘Step-up’ and ‘Treat to Target approach’ and explore how increased insight into the biology of disease may translate into future more targeted strategies for this chronic inflammatory disease at relevant time points: preclinical disease, active disease, and clinically inactive disease.

## Teaser

This review discusses unresolved clinical challenges in juvenile idiopathic arthritis and explores how increased insight into the biology of disease may translate into future treatment strategies for this chronic inflammatory disease at relevant time points: preclinical disease, active disease, and clinically inactive disease.

## Introduction

Juvenile idiopathic arthritis (JIA) is the most common chronic rheumatic disease in children, with a prevalence ranging from 16–150 cases per 100 000 population [1]. JIA is an umbrella term defining several forms of chronic arthritis with an onset before the age of 16 years, persisting for more than six weeks and with an unknown cause [2]. The unifying feature of JIA is chronic arthritis; however its heterogeneity is one of the most intriguing aspects of JIA. Based on the current International League of Associations in Rheumatology classification criteria (ILAR 2003) [3], different subtypes of JIA can be distinguished, essentially by a very limited set of clinical features (number of affected joints in the first six months of disease, extra-articular manifestations like fever or features of psoriasis) and serology (presence or absence of rheumatoid factor, RF). The most frequently diagnosed JIA subtypes are oligoarticular JIA (oJIA), polyarticular JIA (pJIA), and systemic JIA (sJIA). Less frequently occurring subtypes are enthesitis-related JIA, psoriatic arthritis, and undefined arthritis. In this review, we focus on s JIA and nonsystemic JIA (oJIA and pJIA), which are strikingly different disease entities also from a mechanistic point of view.

OJIA refers to arthritis that affects up to four joints within the first six months of the disease and usually involves larger joints, such as knee and ankle. OJIA can be self-limiting in up to 50% of patients [1] but can extend to more than four joints after the first six months and is then referred to as extended o JIA. PJIA arthritis includes five or more joints in the first six months and besides the larger joints, often smaller joints of the hand and feet are involved. PJIA can be accompanied by the presence of RF, an auto-antibody directed against the Fc part of IgG. RF was first described in the 1940s, and in contrast to adult rheumatoid arthritis (RA), only a minority (~5–10%) of pJIA patients have positive titers of RF. Whereas it’s immunological function is still largely unresolved, the presence or absence of RF does affect disease course and outcome in JIA. RF-negative pJIA patients can still have a variable outcome, with some patients achieving long-lasting remission on or even off maintenance therapy, but RF-positive pJIA patients have a very high chance of developing a chronic (lifelong) disease course with a high chance of joint damage as well [4].

One of the most striking extra-articular features of nonsystemic JIA is the occurrence of asymptomatic (silent) anterior uveitis, which occurs in up to 25% of children with nonsystemic JIA subtypes. This association is strikingly different when compared to adult onset chronic arthritis like RA where uveitis is much less prevalent. The incidence of anterior uveitis is highest in antinuclear antibody (ANA) positive JIA patients [5]. As especially young, female oJIA patients are often ANA positive, and this subgroup of patients is yet under debate as a specific disease entity within a revised classification [5].

SJIA is nowadays recognized as a specific subtype of JIA. It is considered an acquired chronic inflammatory disorder with striking autoinflammatory features (i.e. fever, rash, serositis, involvement of neutrophils, monocyte/macrophages, autoinflammatory proteins, etc.). In the early phase of the disease, while autoimmune considered pathways seem to play a role in sJIA patients with a more chronic and/or refractory disease course [6, 7]. sJIA, per definition, displays systemic inflammatory features (fever) with potential organ involvement (skin rash, hepatosplenomegaly, pericarditis, generalized lymphadenopathy, etc.) besides inflammatory arthritis.

Here, we review the main (supposed) mechanisms of disease in both nonsystemic JIA and sJIA. We discuss the rationale behind the most commonly used current strategies: “Step-up” and “Treat to Target” approaches. Moreover, we discuss how mechanisms of disease may translate into future treatment strategies at relevant time points: in occult disease, active disease, clinically inactive disease, and persistently inactive disease. We discuss how current insights into mechanisms of disease and targeted treatment modalities, like biologicals and JAK-STAT inhibitors, resulted in significantly improved outcomes and reduced risks for articular damage. We also discuss how loss of immune tolerance and subsequent development of chronic inflammation in these children is still inadequately understood and results in ongoing inflammation while on treatment in a considerable number of patients, referred to as refractory JIA. These patients still face significant health challenges, uncertain long-term outcomes and, therefore, a major burden on their daily life in childhood and beyond.

## Immunopathology in nonsystemic and sJIA

### Etiology of nonsystemic and sJIA

The cause of and underlying etiopathogenetic mechanisms in juvenile idiopathic arthritis are still largely unknown and assumed to be multifactorial: genetic, epigenetic, and environmental.

#### Nonsystemic JIA:

Genome wide association studies (GWAS) have shown that multiple genes and risk alleles may contribute to the risk of developing oJIA and pJIA in childhood. Clear associations with HLA-related genes and non-HLA -elated loci have been reported, including *PTPN22, PTPN2, IL2, IL2RA, STAT4, RUNX1*, strongly suggesting that T-cell dysregulation and activation is a major factor in the pathogenesis of JIA [8–12]. It is currently believed that a genetically susceptible individual develops a deleterious and uncontrolled response toward one or more self-antigens on exposure to an unknown environmental trigger (-s). Epigenetic mechanisms like methylation or histone modification act at the interface between disease risk factors (including environmental factors, nutrition, infection, etc.) and the implementation of the genetic information encoded in DNA. For JIA, epigenetic analysis of specific histone marks (H3K27ac) showed that synovial fluid derived CD4+ T cells and monocytes display a disease-specific signature of both enhancers and super-enhancers, non-coding regulatory elements in *cis*-acting DNA sequences of several hundreds to up to 50 000 (50 kb) base pairs in size, to which transcription factors and cofactors can bind and control transcription. Interestingly, “treating” patient-derived T cells *in vitro* with the BET-inhibitor JQ1 resulted in a preferential inhibition of genes that were upregulated in JIA [13, 14].

Contributing factors associated with the occurrence of JIA are among others bacterial infections and use of antibiotics in the first year of life [15, 16]. Interestingly, the initial pattern of affected joints has also been suggested to influence disease outcome in JIA [17]. Joint inflammation in nonsystemic JIA is characterized by accumulation of activated memory T cells in the synovium, which are clustered around antigen-presenting (dendritic) cells [18]. Besides T and B cells, also innate cells such as macrophages and fibroblasts become activated and migrate into the synovium lining of the joint. Upon activation, these cells start to produce cytokines and express adhesion molecules that allow for continued ingress of immune cells. Thus, the initial triggering response seems to result in activation of both innate and adaptive immune pathways that causes tissue damage resulting in the release of excessive concentrations of self-antigens in the joint. Responses to self-antigens have been reported and might be of clinical importance for disease course, but might also hold promise for therapeutic strategies [19] as epitope-specific T cell clones from the “self-derived” damage-associated protein *HSP60* have a tolerogenic signature [20]. Tissue-resident effector memory T cells (Trem) in the joints also seem to play a role in the remitting-relapsing course that is typical for nonsystemic JIA [21]. Intriguingly, JIA flares preferentially affect previously inflamed joints or show a pattern of laterality suggesting joint-specific memory [22, 23]. The exact mechanisms for this joint-specific memory remain still largely unknown, but a role for Trem cells and synovial fibroblasts was suggested [22, 24]. Altogether, the chronicity of a self-perpetuating loop in nonsystemic JIA-induced inflammation seems to result from the inability of regulatory mechanisms to suppress excessive effector mechanisms [1].

#### SJIA:

This JIA subtype hallmarked by profound systemic inflammation is still classified under the umbrella of JIA. However, evidence is accumulating that at least in the initial and early phases of sJIA the mechanisms of disease differ significantly from mechanisms of disease in nonsystemic JIA [10]. On a genetic level, single-cohort studies using a candidate gene approach to examine small case–control collections found associations of sJIA with SNPs in mostly innate immune-related genes, including genes encoding cytokines and cytokine receptors [25–27]. A recent GWAS study involving 770 sJIA patients from nine countries showed that sJIA is rather dissimilar to nonsystemic JIA, and revealed two novel and 23 previously described risk loci [10]. Interestingly, this GWAS study also showed a clear association with an MHC locus on chromosome 6, suggesting a contributing role of the adaptive immune system. In succession of this effort, the NIH group published a follow-up analysis of their international gathered cohort (the same 770 patients) in 2018 [28]. Here, extended genetic analysis showed that the only region to be significantly associated with sJIA was on chromosome 1, located in the promoter region of *IL1RN*, the gene encoding for the IL-1 receptor antagonist. Currently, there are no data on specific epigenetic signatures of or in sJIA available in literature. Suggested environmental etiological factors for sJIA are, e.g. (seasonal) infections [29].

Indeed, new onset sJIA patients generally display-marked features of autoinflammation, with a predominance of disturbed innate immune mechanisms. sJIA patients display marked neutrophilia, features of monocyte/macrophage involvement, IL-1 pathway activation and increased plasma levels of IL-6 and IL-18, all indicative of a prominent autoinflammatory type inflammation [6]. So far, no specific auto-antibodies have been reported in this disease. Increased insight in the autoinflammatory characteristics of this specific JIA subtype over the past decade has been successfully translated into clinical practice, with the registration of both IL-1 and IL-6 blockade for use in sJIA in both the US and Europe. Indeed, early targeted treatment with recombinant IL-1receptor antagonist (rIL-1RA, anakinra) has resulted in strikingly high response rates in sJIA [30–32]. Although more than 50% of anakinra-treated patients achieve remission and can stop rIL-1RA therapy within one year [32], other sJIA patients fail to completely respond to IL-1 blocking therapy necessitating the concomitant use of glucocorticoids and/or switch to IL-6 blocking therapy [33, 34]. Around 30% of sJIA patients develop a refractory disease course, with, e.g., refractory arthritis, recurrent episodes of macrophage activation syndrome (MAS) or the development of interstitial lung disease [7]. Refractory sJIA has been associated with the involvement of adaptive immune disease mechanisms, which was also supported by genome wide association studies that link-specific HLA alleles with this disease [7, 10, 35, 36].

### Relevant disease mechanisms in nonsystemic and sJIA

**Figure 1.**
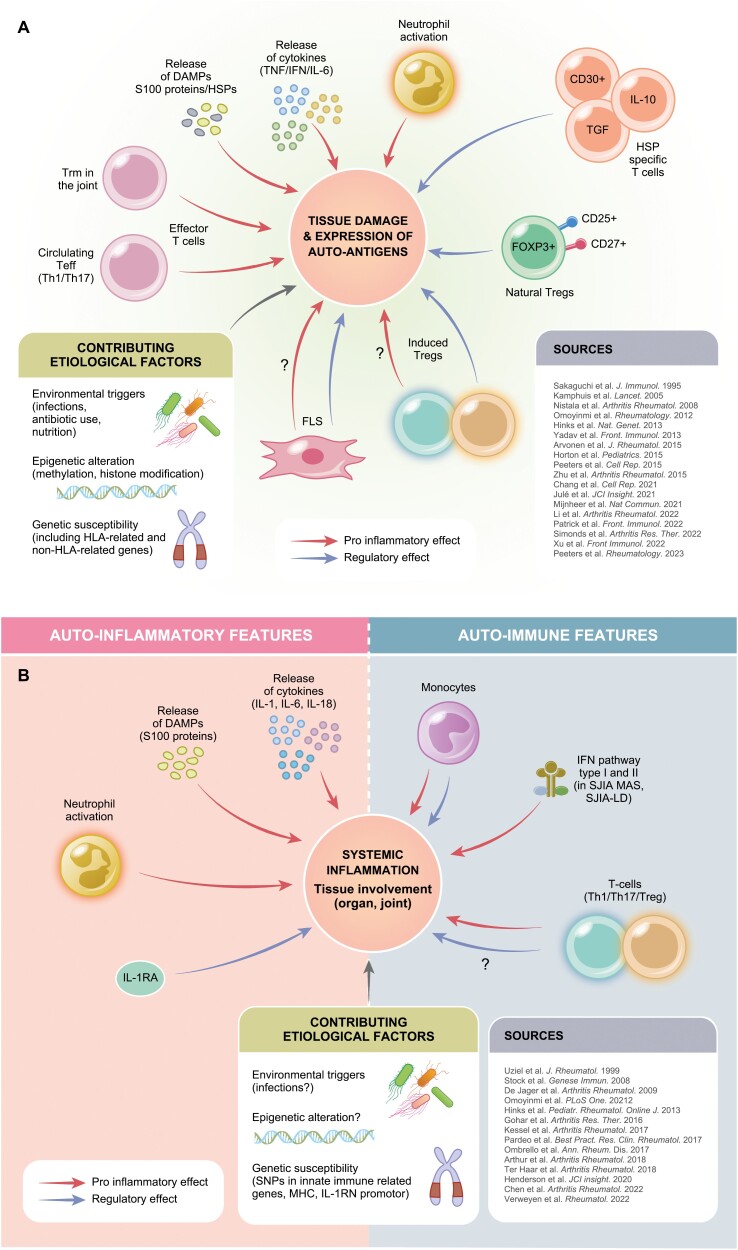
Disease contributing mechanisms (both effector and potentially regulatory) in non-systemic JIA (Fig. 1a) and sJIA (Fig. 1b). Modified from Prakken et al., PMID 21684384 [1].

One of the most intriguing aspects of this set of chronic inflammatory diseases, is its heterogeneity and its subtype-specific/extra-articular disease manifestations like the high prevalence of anterior uveitis in nonsystemic JIA and the extra-articular involvement with spiking fevers, skin rash, and organ involvement in sJIA. We are only recently beginning to understand that sJIA and nonsystemic JIA seem to differ in their main effector mechanisms and possibly also in the (lack of) regulatory mechanisms involved. These are summarized by and large in Fig. 1 (Fig. 1a for non-systemic JIA, Fig. 1b for sJIA). Whether these different disease mechanisms also result in the different clinical phenotypes or not and how these evolve in refractory or no-refractory disease courses is still only partly understood. In any case, a deeper understanding of specifically the involved effector mechanisms in different JIA subtypes has driven the development and use of more specific or targeted therapy modalities in both nonsystemic and sJIA [6, 37].

### Regulatory mechanisms in oligoarticular and polyarticular (nonsystemic) JIA

The clinical reality that JIA can have a remitting course as well as a chronic disease course, has led to the assumption that there is a distortion in control of regulatory immune processes over effector cells/mechanisms leading to a loss of immune homeostasis and chronic inflammation directed to, or maintained by, self-antigens in the joints. Regulatory T cells (Treg), first described by Sakaguchi [38] constitute a unique population of T cells that can specifically suppress other immune cells in proliferation or action (like the production and excretion of cytokines). There are multiple Treg subsets: *Natural* Treg cells develop in the thymus, constitutively express the forkhead transcription factor *FoxP3* and subsequently execute their function in peripheral (lymphoid) tissues [39]. These cells seem to have a crucial role in maintaining immune homeostasis and in preventing the development of autoimmune responses. *Induced* Treg cells comprise of different subtypes of cells, derived from CD4 + T cells in the periphery, including (but not limited to) interleukin 10 (IL-10)-producing Tr1 cells and transforming growth factor-β (TGF-β)-producing T cells [40]. Multiple translational studies have shown that within the synovial fluid, Treg numbers are increased, not decreased. Although these Treg seem functional in suppressing peripheral blood-derived effector cells, T effector (Teff) cells from the site of inflammation seem to be resistant toward Treg-mediated control [41, 42]. Recent studies indicate that the specific environment (like the inflammatory joint space) is able to induce further Treg adaptation into specialized activated Treg subsets, now referred to as effector (e)Treg [43, 44]. Indeed, inflammation-derived Treg cells acquire a conserved and specific eTreg cell profile guided by epigenetic changes, and fine-tuned by environment-specific adaptations [45]. Where early studies on Treg primarily focused on their role in maintaining immune tolerance, it is currently understood that tissue-localized and resident Treg are also important modulators of tissue protection and repair [46–48].

### Effector mechanisms in oligoarticular and polyarticular (nonsystemic) JIA

Different T helper (Th) cell subsets have been implicated as effector cells in the pathogenesis of JIA [49]. Although in peripheral blood there are no differences found in CD4+ and CD8+ T cells subsets between JIA patients compared to controls, they do respond differently to Th1 and Th17 polarizing conditions with an increased production of the proinflammatory cytokines IFNy and IL-17 [50]. In synovial fluid from inflamed joints of JIA patients, a mixed Th17/Th1 phenotype is found and the presence of this subset also correlates with disease activity [51]. Th1 cells have been implicated in disease development and progression [18, 52]. Furthermore, specific CD4+ Th cell subsets show differential cytokine expression between the subtypes of JIA implying a role for Th cell subsets in disease pathogenesis [53]. Most prevalent T cells in SF are CD4+ and CD8+ effector T cells with an effector memory T cell phenotype (T_EM_) [49, 54]. Synovial T_EM_ are highly activated and more oligoclonal compared to their blood counterparts [49, 55–57]. However, Treg which are also abundant in SF compared to peripheral blood in JIA patients are unable to effectively suppress effector cells at the site of inflammation in JIA. Although these Treg have kept their suppressive capacity in coculture experiments with peripheral blood-derived effector cells, interestingly enough, it seems that both CD4+ and CD8+ effector T cells have become resistant (both in numbers and cytokine secretion) to regulation by autologous Treg [58]. This could however be reversed *in vitro* by Tumor Necrosis Factor alpha (TNFα) blockade [59, 60].

An increased CD8/CD4 ratio in the SF has been shown to be predictive for developing a more severe JIA disease phenotype [61]. This is thought to be promoted by SF CD8+ T cells producing high levels of the CD8+ T cell chemo-attractant CCL5 (RANTES), thereby inducing a positive feedback loop for CD8+ T cell recruitment.

Next to T cells, there are other cells abundantly present in JIA-derived-SF. Neutrophils, which may have an altered phenotype and effector function in the SF compared to peripheral blood [62–64], and switched memory B cells, which may function as antigen presenting cells in the joints of patients with JIA [65, 66]. Furthermore, fibroblast-like synoviocytes (FLS) from JIA patients express a heterogeneous gene signature between nonsystemic JIA subtypes [67], which implies a relevant and distinguishing role for FLS in nonsystemic JIA pathogenesis.

### Effector and regulatory disease mechanisms in sJIA

Various translational cohort studies demonstrated that the early phase of sJIA is characterized by autoinflammation and sustained by activation of the IL-1 and IL-18 pathway [68–70]. Indeed, a prominent feature of sJIA is the marked neutrophilia at disease onset [71, 72]. These neutrophils display a primed phenotype and a sepsis-like immune signature and secrete relevant amounts of S100A8/9 and S100A12 proteins providing a positive feedback mechanism in sJIA with subsequent IL-1 and IL-18 production [73]. Another feature of sJIA is defective NK cell function (and lower numbers in peripheral blood), suggested to contribute to the increased risk for developing MAS, a rather prevalent and dangerous complication of sJIA. [68, 74]. Monocyte/macrophages seem to play a complex (dual) role in active sJIA, with both disease contributing as well as potential counteracting roles with respect to the developing systemic inflammation [75–77]. Intriguingly, there is a clear HLA association in sJIA, suggesting a prominent role for adaptive immune disease mechanisms as well [35]. It seems that especially a refractory disease course in sJIA, defined as either chronic or ongoing arthritis despite maintenance treatment with targeted biologicals [78, 79], recurrent MAS episodes or the development of interstitial lung disease [80, 81], is associated with specific T cell subset involvements [7]. Henderson et al. showed that patients with acute sJIA exhibited increased activation of Treg cells with a Th17 gene expression signature, while patients with a more chronic disease course seemed to have undergone a shift from Treg to Teff cells with a Th17 signature [78]. The involvement of IL-17 producing cells in sJIA was also confirmed by Kessel et al. [79].

In the early phases of sJIA, the role of the innate regulatory protein “IL-1Receptor antagonist” seems crucial in the disease. The *IL-1RN* gene, encoding natural occurring IL-1RA, is a risk locus for sJIA, and variations in the promotor associated with a relatively low expression of IL-1RA seem to associate with an increased risk for developing sJIA. From a therapeutic and translational point of view, rIL-1RA has been proven a highly effective therapy for many patients with sJIA, especially when started early in the disease course [32, 82].

### Current treatment strategies in JIA

#### Targeting effector versus regulatory pathways

The therapy for both nonsystemic and sJIA has been revolutionized by the introduction of biological therapies in the early 2000s. Since then more than a dozen of targeted, antibody-mediated, drugs have been registered for use in pediatric arthritis. This has resulted in a significantly improved outcome on the short and mid/long term for both nonsystemic and sJIA [4]. It is interesting to note that all of the currently registered biological drugs target effector molecules or cells, resulting in downregulation or blockade of immune effector pathways (see Fig. 1a and 1b). Current therapeutic modalities do not directly target regulatory mechanisms in JIA.

##### Antibody-mediated agents in nonsystemic JIA.

The most widely used biological therapies include TNFα-blocking agents (both directed at TNFα itself or at the receptor level) and IL-6 blocking agents (tocilizumab, blocking the IL-6 receptor) [83, 84]. In addition, abatacept (CTLA-4 Immunoglobulin) can be used in pJIA [85, 86], working as a T cell activation blocker by selectively binding to both the CD80 and CD86 receptor of antigen presenting cells, resulting in less activation of effector T cell populations.

##### Antibody-mediated agents in sJIA.

Both IL-1 (short-acting anakinra and long-acting canakinumab) and IL-6 blocking (tocilizumab) agents have been registered for use in sJIA. Anakinra is the recombinant protein of IL-1 receptor antagonist, blocking the IL-1 receptor for binding to both IL-1α and IL-1β. It has a short half-life (4-6 hours) and needs to be injected every day. Canakinumab is the high-affinity blocking antibody to IL-1β, blocking the IL-1 pathway at the level of the cytokine itself. Interestingly, IL-6 blockade by tocilizumab has a rather broad direct effect on both innate cells and T cells. IL-17 targeting biologicals are currently being trialed in sJIA, specifically in those patients with chronic arthritis. These agents are already approved for the specific JIA subtypes psoriatic arthritis and enthesitis-related arthritis (beyond the scope of this review). Antibodies targeting IL-18 are in development.

As stated, these biological therapies all directly target/inhibit effector pathways, affecting both adaptive as well as innate immune cell subsets [87, 88] In addition, neutralizing TNF- or IL-6 pathways may also have an indirect effect on regulatory mechanisms like Treg development or suppressive capacity [89, 90].

##### JAK-STAT inhibition.

More recently and derived from trials and studies in the adult counterparts of pediatric arthritis (like RA), the JAK-STAT inhibitors have been introduced in pediatric rheumatology. JAK-STAT inhibitors are small molecules that have the advantage over biologicals of being administrated orally (and not intravenously or subcutaneously). So far, only tofacitinib, a JAK-STAT 1-2-3 pathway inhibitor, has been registered for use in pJIA [91]. JAK-STAT inhibition diminishes the phosphorylation of activated cytokine receptors, directly affecting the transcription of several cytokines and thereby the activation of (multiple) cytokine pathways including type I and II interferons. Tofacitinib is known to block the production of IFNγ, IL-2, IL-4, IL-6, IL-15, and IL-21, thus affecting both Th1 and Th2 T cell populations, and again most prominently affecting effector pathway-derived molecules and cells, not so much regulatory mechanisms directly. Tofacitinib is currently being trialed in sJIA as well (NCT03000439), for the treatment of systemic features of the disease. Moreover, it has been suggested to be helpful in refractory course sJIA [7].

#### “Step-up” treatment approaches versus “Treat to Target” approaches

As discussed, the arsenal of registered therapies in nonsystemic and sJIA has significantly grown in the past two decades. Nevertheless, JIA is still treated by a “step-up” approach in most Western countries, in which methotrexate (MTX) is the mainstay of initial treatment in nonsystemic JIA. For sJIA, the first-line therapy used to be MTX combined with high dose glucocorticoid treatment, although targeted treatment with (short acting) IL-1 receptor antagonist anakinra is increasingly being used as first-line therapy.

In nonsystemic JIA, patients that do not respond well enough on MTX as starting maintenance treatment are often only recognized after several months of treatment. These patients are subsequently treated with biologicals, with generally TNFα blockers as the first class of biological therapy. There is an advantage in combining TNFα blockade with MTX over TNFα monotherapy [92]. In patients refractory to both MTX and TNF blockers, IL-6 blockade, abatacept, or tofacitinib can be used, most often also in combination with MTX. Current literature does not provide evidence for a preferred third-line treatment in nonsystemic JIA, and therefore, current treatment guidelines do not give preference on one specific biological therapy after failure on both MTX and TNF blockade.

Given the disadvantages of the “step-up” approach and based upon high response rates to biological therapies even in patients that are unresponsive to MTX, many treating physicians have adopted a “Treat to Target” (T2T) strategy for the treatment of JIA in the past five years [93]. This strategy strives to achieve targeted treatment responses at given time points in the disease and is more flexible in the order and combination of available therapeutic drugs (e.g. MTX combined with another DMARD and/or biologicals from the start of treatment). Moreover, this strategy promotes close monitoring of JIA patients, arguably resulting in quicker modification of the treatment and a swifter clinical improvement in many JIA patients. A striking example of a T2T strategy in JIA is the Best 4 Kids study, comparing three treatment arms (DMARD only, MTX combined with corticosteroids, and MTX combined with anti TNF) but also allowing early “crossover” to the combination arm of MTX combined with anti-TNF when the treatment target at time point three months was not met [94].

Another example, now for sJIA, is first-line treatment of SJIA with rIL-1RA, avoiding the use of glucocorticoid treatment in >75% of new onset patients and resulting in high response rates as well as the ability to taper and stop biological maintenance therapy in >50% of sJIA patients within the first year of disease [32].

These first T2T studies show encouraging effects on treatment responses and potentially disease outcomes. However, the current mechanistic insights and available literature do not provide sufficient support to biologically stratify JIA patients to a specific biological therapy or JAK-STAT inhibitor. The consensus treatment plans (CTP) from, e.g. the US Childhood Arthritis & Rheumatology Research Association (CARRA) [95] and the German Society for Pediatric Rheumatology (GKJR) [96] as well as (inter-)national translational cohort studies may provide useful insights into the preferential first choice of biological treatment in the future. When patients are immunologically characterized prior to the start of therapy, we may learn to associate relevant activated biological immune pathways at disease or therapy onset to outcome of specific therapeutic modalities [97].

#### Unresolved questions in the current treatment era and future strategies

**Figure 2.**
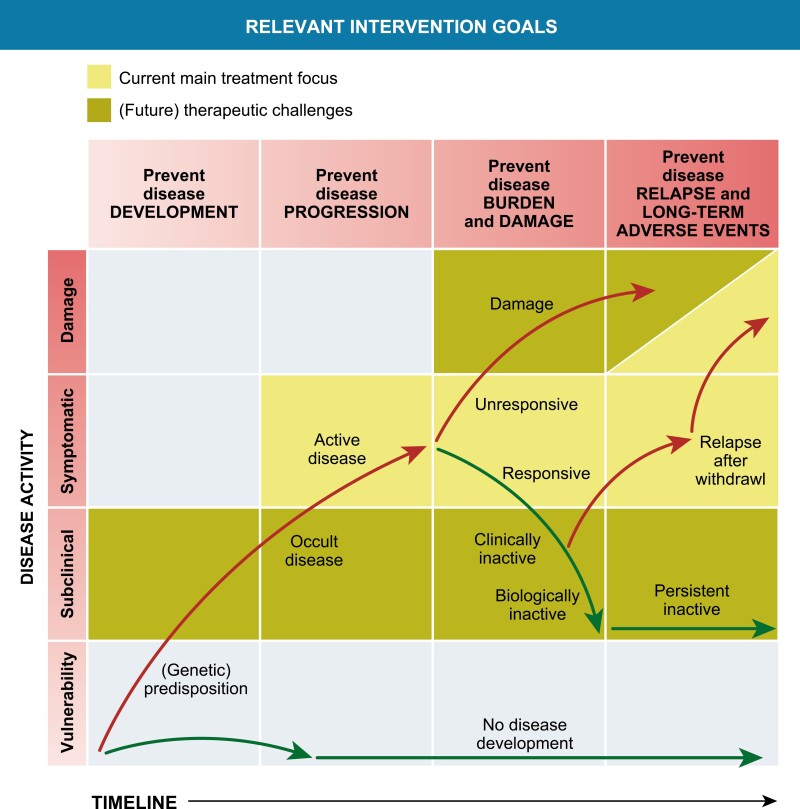
Relevant intervention opportunities in the development and treatment of chronic inflammation in JIA. Modified from Swart et al., PMID 27461267

In the past two decades, the development of biological DMARDS and their registration for specific JIA subsets have significantly improved the outcome of patients with JIA. However, there is still a significant subgroup of JIA, both in nonsystemic as sJIA, that need to cope with the challenges of having *refractory* JIA. These children often still require systemic glucocorticoids as part of their treatment and as such are at risk for long-term damage of active disease as well as the side effects of treatment. In the current treatment era, there are important remaining prevention and treatment challenges, schematically addressed in Fig. 2.

##### Strategies to prevent the onset of chronic articular inflammation in high-risk patients.

 Preventing the development of a chronic disease course in those children who are at risk is of course an ultimate goal for clinicians and researchers. In adult RA, multiple studies have shown that RF and anti-citrullinated protein antibodies (ACPAs) are detectable in serum of patients months to years before disease onset [98]. These observations, when accompanied by translational studies providing insight in contributing factors to the development of chronic arthritis in these individuals, open up a “prevention window,” to develop strategies to minimize or even prevent the subsequent onset of clinically apparent articular disease. Unfortunately, in JIA, diagnostic biomarkers/strong risk factors for preclinical disease have not yet been found or validated.

##### Toward personalized treatment regimens.

As discussed previously in this review, the choice of biological therapy or start of JAK-STAT inhibition is currently not based upon insight in the patient’s most active or relevant immunological pathways. It is currently a matter of physician or patient’s preference how to proceed when a patient flares after unsuccessful treatment with MTX and a first biological (most often anti-TNF as first-line biological therapy in nonsystemic JIA). To further improve disease outcome in non-systemic and sJIA, it could be very relevant to base the choice of therapy upon biologically active immune pathways. For RA, this has already been shown to hold promise [99]. For JIA, a new, more biologically based classification of JIA might be required [100] and large scale translational inception cohort studies are necessary to provide insight into the relevant active immune pathways both in those patients that show a good response to initial MTX therapy, and in those that need biological therapy. Currently, multiple of these cohort studies are underway (e.g. www.ucancandu.com and CLUSTER) [101]. These studies could provide insight into whether it is feasible to change the biological course of chronic arthritis. Indications this goal is feasible in some patients can be deducted from studies in the late 90s and early 2000, in which autologous stem cell transplantation for JIA patients unresponsive to all, at that time, available therapy, induced a long-lasting disease remission off medication in about first/second of patients [102].

##### Improving tapering and stop strategies in clinically inactive disease on maintenance treatment.

A third unresolved clinical challenge is how to proceed in children that do achieve a state of clinically inactive disease. Nowadays, we can achieve a state of clinically inactive disease while on treatment in a substantial number of our patients. The question is whether this state is mere disease suppression to a level that is clinically not detectable, or whether this is the result of changing the biology of the disease course and thereby a first step toward a “cure” in a subset of patients. Currently, we are developing strategies to taper and stop maintenance treatment based upon experience and patients preference. Even without the occurrence of irreversible joint damage through improved treatment, at this moment, it is still unclear what the effects of continuous treatment with MTX or biologicals for 5, 10, or even more years are on the long-term well-being of our patients. Does this affect, e.g. fertility? Or does this have an impact on the risks for cardiovascular events? Or even the risk of developing a malignancy? There is some guidance from observational cohort studies for tapering biological therapy and one hallmark study that randomized patients toward a rather quick (nine months after achieving clinically inactive disease upon MTX treatment) or a slower tapering path (start tapering 15 months after achieving clinically inactive disease) [103–105]. These studies show that the risk of flare in patients in clinically inactive disease on treatment that start tapering and stopping maintenance therapy is as high as 50–85% in the first 1–2 years after stop. Validated biomarkers indicative of subclinical disease activity in both nonsystemic and sJIA will be of major help in optimizing tapering and top strategies. These have recently been thoroughly reviewed in 2016 and 2021 [106, 107]. Promising biomarkers in nonsystemic and sJIA like the S100/MRP proteins (S100A12 and S100A8/9), IL-18, and others are currently being validated [108–110]. We need (commercialized) cost-effective implementation strategies before these will affect clinical practice in general.

#### Focus on strengthening immune regulatory mechanisms

Another strategy could be to develop ways to strengthen or improve regulatory mechanisms in children that have successfully responded to strategies directed at effector pathways (i.e. biological therapy or JAK-STAT inhibition). Possible venues to explore are antigen-specific tolerization that has been trialed in RA a decade ago [19]. An attractive option is to explore strategies directed at improving number and function of Tregs, e.g. by starting nicotinamide maintenance therapy in patients that achieved clinically inactive disease. Nicotinamide has proven to be safe for maintenance therapy in high doses [111, 112] and seems to be able to increase both the number and function of Tregs [113] (Nijhuis L. et al., unpublished observations).

## Conclusion and future directions

In this review, we summarized relevant mechanisms of disease in both nonsystemic and sJIA, with a focus on how these mechanisms are targeted by current therapy modalities. We indicated several unresolved clinical questions and challenges, and suggested ways to potentially tackle these. Even in the current era, with multiple, and even increasing, modalities to target chronic inflammation in childhood arthritis, we should not be satisfied with the progress of the past decade. Instead, we need to reset our goals and focus on developing biologically stratified treatment strategies with the currently available drugs. Moreover, there is a need to optimize tapering of treatment when possible as well as developing less intensive maintenance therapies. This might involve combining therapeutic modalities early in the disease course to really modulate immune responses and change the biology of the disease, and then step down in therapy when clinically inactive disease criteria are met. As valuable will be, therefore, the development of therapeutic maintenance modalities that are theoretically less risky, directed at strengthening immune function, not suppressing.

## Data Availability

Not applicable.

## Abbreviations

ACPAs: anti-citrullinated protein antibodies
ANA: antinuclear antibody
CTP: consensus treatment plans
FLS: fibroblast-like synoviocytes
GWAS: Genome wide association studies;
JIA: juvenile idiopathic arthritis
MAS: macrophage activation syndrome
MTX: methotrexate
sJIA: systemic juvenile idiopathic arthritis
oJIA: oligoarticular juvenile idiopathic arthritis
pJIA: polyarticular juvenile idiopathic arthritis
RF: rheumatoid factor
T2T: treat to target
Th: T helper
Treg: regulatory T cells
Trem: tissue-resident effector memory T cells

## Clinical Trial Registration

Information on clinical trial registration is cited in the text where applicable.
